# Function of IRAG and the phosphorylation of the InsP_3_R-I for the NO/cGMP-dependent inhibition of platelet aggregation

**DOI:** 10.1186/1471-2210-11-S1-P58

**Published:** 2011-08-01

**Authors:** Katharina Salb, Elisabeth Schinner, Jens Schlossmann

**Affiliations:** 1 Department of Pharmacology and Toxicology, University of Regensburg, Germany

## Background

Precondition for activation and aggregation of platelets is a rise in intracellular calcium concentration which can be inhibited by activation of the NO/cGMP/cGKI signalling cascade. The cGMP-dependent Kinase I (cGKI) is assembled in a macrocomplex with the Inositoltrisphosphate receptor I (InsP_3_R-I) and the Inositoltrisphosphate receptor associated cGMP kinase substrate (IRAG).

## Results

We investigated the relevance of IRAG and the cGKI stimulated phosphorylation of the calcium channel InsP_3_R-I for the NO/cGMP-dependent inhibition of platelet aggregation and adhesion.

After incubation with different agonists (collagen, thrombin, TxA2) we performed aggregation experiments with platelets of WT and IRAG-KO mice, thereby the IRAG-KO platelets aggregated stronger than the WT platelets. After preincubation with NO/cGMP the inhibition of aggregation was decreased in IRAG-KO platelets compared to WT platelets. Furthermore, GPIIb/IIIa-mediated adhesion of platelets to fibrinogen could only weakly be inhibited in IRAG-deficient platelets contrary to WT platelets. The cGKI-mediated stimulation of InsP_3_R-I phosphorylation showed an equal increase in WT and IRAG-KO platelets.

## Conclusion

These results revealed that IRAG plays an important role in the NO/cGMP-dependent inhibition of platelet aggregation. However, the cGMP-stimulated phosphorylation of InsP_3_R-I is not necessary for the inhibition of platelet aggregation.

